# Gait characteristics in people with Friedreich ataxia: daily life versus clinic measures

**DOI:** 10.3389/fneur.2025.1544453

**Published:** 2025-03-17

**Authors:** Hannah L. Casey, Vrutangkumar V. Shah, Daniel Muzyka, James McNames, Mahmoud El-Gohary, Kristen Sowalsky, Delaram Safarpour, Patricia Carlson-Kuhta, Christian Rummey, Fay B. Horak, Christopher M. Gomez

**Affiliations:** ^1^Department of Neurology, The University of Chicago, Chicago, IL, United States; ^2^APDM Wearable Technologies - A Clario Company, Portland, OR, United States; ^3^Department of Neurology, Oregon Health & Science University, Portland, OR, United States; ^4^Department of Electrical and Computer Engineering, Portland State University, Portland, OR, United States; ^5^Clinical Data Science GmbH, Basel, Switzerland

**Keywords:** gait, free-living, Friedreich’s ataxia, wearable inertial sensors, digital biomarker, clinical trials

## Abstract

**Introduction:**

Gait assessments in a clinical setting may not accurately reflect mobility in everyday life. To better understand gait during daily life, we compared measures that discriminated Friedreich ataxia (FRDA) from healthy control (HC) subjects in prescribed clinic tests and free, daily-life monitoring.

**Methods:**

We recruited 9 people with FRDA (median age: 20, IQR [12, 48] years). A comparative healthy control (HC) subject cohort of 9 was sampled using propensity matching on age (median age: 18 [13, 22] years). Subjects wore 3 inertial sensors (one each foot and lower back) in the laboratory during a 2-min walk at a natural pace, followed by 7 days of daily life. For daily life analysis, a total of 99,216 strides across 1,008 h of recording were included. Mann–Whitney U test and area under the curve (AUC) compared gait differences between FRDA and HC when assessed in the laboratory and daily life. Pairwise Wilcoxon tests also compared if participants exhibited different metric values between the two environments.

**Results:**

The FRDA group exhibited lower levels of daily activity. Measures that best discriminated gait characteristics of FRDA from HC differed between environments. Variation in elevation of the feet at midswing best discriminated in-clinic (Clinic AUC = 1, Home AUC = 0.69), whereas slow gait speed performed best in daily life (Home AUC = 1, Clinic AUC = 0.64). Of the 17 measures tested, 11 had an AUC > 0.8 in-clinic and 8 had an AUC >0.8 at home. Variability of swing time (Clinic AUC = 0.97, Home AUC = 0.94) and double-support time (Clinic AUC = 0.94, Home AUC = 0.94) were the most sensitive and specific for FRDA in both environments.

**Conclusion:**

Digital gait characteristics from inertial sensors are sensitive and specific for FRDA in both environments. However, different gait measures were more sensitive and specific during free-living versus prescribed gait, suggesting that in-clinic gait does not reflect daily life gait.

## Introduction

Progressive gait impairment is one hallmark feature of Friedreich ataxia (FRDA) (1, 2). As interventional therapies for FRDA emerge, there is a growing need for quantitative gait assessments that measure disease severity and progression. Previously, quantitative measures of gait were only accessible via prescribed walking tests in the laboratory or clinic under controlled conditions. Although these tests provide valuable insights about gait impairments under ideal conditions, they may not reflect real-life gait performance during everyday activities (3–5). Performance on the walking tasks in a clinic or laboratory setting may be enhanced due to the Hawthorne effect of being observed by a physician or researcher, whereas in real-life scenarios, distractions and varying conditions and environments may contribute to worsened gait performance. Therefore, gait assessments in clinical and laboratory settings measure an individual’s ideal performance capabilities, whereas gait in daily life reveals their practically relevant abilities (4, 5).

Advancements in wearable technologies have enabled the precise measurement of gait in both the clinic setting and in daily life for many neurological conditions including Parkinson’s disease (6–21), multiple sclerosis (7, 22–24), Huntington’s disease (12), cerebral palsy (25), and degenerative cerebellar ataxia (26). However, only a few studies have evaluated wearable technology used in the daily life monitoring of gait characteristics of FRDA (27–29).

In-clinic assessments have many limitations in FRDA cohorts that may be addressed with daily life measurements. Given the limited number of FRDA specialists, there are great barriers to clinical care and participation in clinical research studies. Accessing these resources often necessitates lengthy and costly travel to the few available specialized clinics. These challenges are further complicated by the need to navigate the school and recreational schedules of young individuals diagnosed with FRDA, as well as the complexity of participating in clinical drug trials. In-home data collection of gait measures can provide a more cost-effective and schedule-conscious approach to assessing an individual’s condition. By deploying wearable devices in daily life settings, home monitoring may provide a more comprehensive and continuous understanding of disease severity, disease progression, and treatment efficacy in FRDA populations.

The purpose of this study is to identify the gait measures that best discriminate between individuals diagnosed with FRDA and age-and sex-matched healthy controls (HC) from a 2-min walking test at a natural pace in the clinic using wearable inertial sensors. We compared these prescribed task measures to gait measures collected over a week of free-living activity from daily life. We explored whether the gait measures that most sensitively and specifically distinguish gait impairments in FRDA from HC during in-clinic settings are the same as those assessed in daily life. We hypothesized that: (1) distinct gait measures would best discriminate FRDA from HC in clinical and daily life settings, and (2) gait characteristics would differ between clinic and daily life in the same subjects tested.

## Materials and methods

### Ethics approval statement

This study was approved by the University of Chicago BSD IRB and Oregon Health & Science University IRB Committees (reference number 18-1580 and 21,082, respectively). Written informed consent and assent was obtained from patients to participate in an observational study to investigate disease severity and progression.

### Participants

We recruited adult and pediatric participants with FRDA and age-matched HC as part of a larger study (IDEA study) (30, 31). FRDA participants who performed in-clinic assessments at designated sites were also given the chance to enroll in at-home daily life monitoring (*n* = 12). Inclusion in the larger study was limited to participants who were able to walk independently 10 meters for up to 2-min and sit unassisted for 30 s, with supervision. Early-onset and typical-onset FRDA participants were those aged between 12 and 30 years old and diagnosed between the ages of 5 and 25. Late-onset FRDA participants were those aged between 26 and 60 and diagnosed between the ages of 25 and 40. Exclusionary criteria include any history of head injury, vestibular dysfunction, stroke, or other neurological condition or musculoskeletal disorder that could affect mobility.

### In-clinic data collection

**Figure 1:**
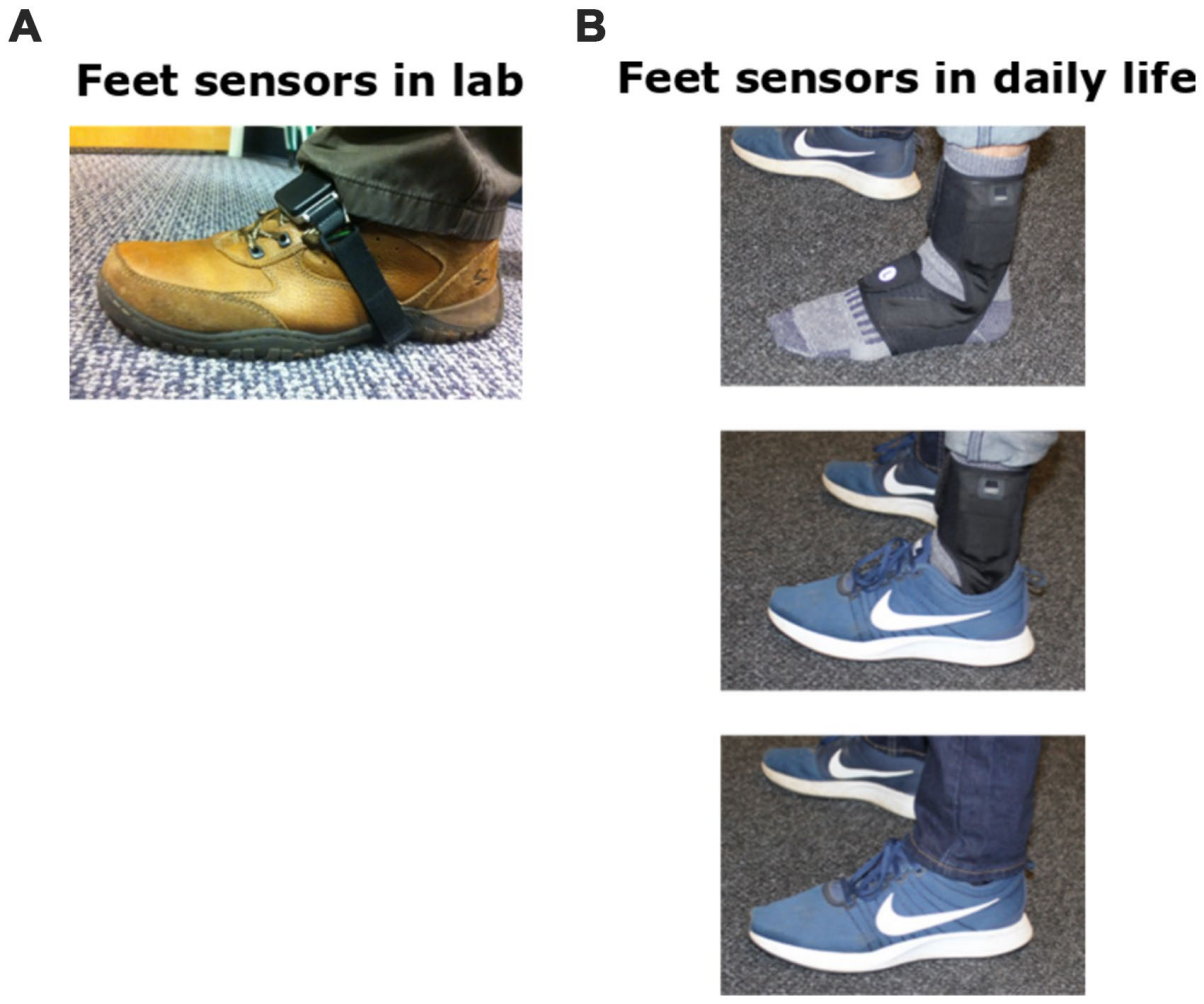
Wearable sensor placement on feet. **(A)** Opal sensor on foot for clinic or laboratory testing. **(B)** Instrumented sock for at-home daily life collection of gait.

In the clinic, subjects were asked to wear 6 inertial sensors (Opals by APDM Wearable Technologies, Portland, OR, United States; Figure 1A), one sensor on dorsum of each foot, one sensor on dorsum of each hand, one sensor over the sternum area, and one sensor over the lower lumbar on an elastic belt. Each Opal sensor includes a tri-axial accelerometer, gyroscope, and magnetometer with a sample rate of 128 Hz. Subjects completed the 2-min walk test (2MWT) over a 10-meter pathway, as part of a larger battery of tests. Subjects were assessed using the Scale for the Assessment and Rating of Ataxia (SARA) (32) and Modified Friedreich’s Ataxia Rating Scale (mFARS) (33). The Upright Stability Score (USS) and total Neurological Exam (NE) scores were calculated from the mFARS exam. Patient-reported FARS – Activities of Daily Living (ADL) (34, 35) were also collected. We only used 3 sensors (2 on each foot and 1 lumbar) for gait characteristics from in-clinic to compare with daily life gait characteristics.

### Daily life gait data collection

To reduce the burden on participants of trying to securely attach the Opal monitors to the outside of their shoes, APDM Wearable Technologies designed an instrumented, neoprene sock (Figure 1B) that wraps around the participant’s foot and ankle with the same inertial sensors inserted into a small, lightweight pocket on the dorsum of the foot. The battery is located in a second pocket just above the lateral malleolus. The inertial system fits into the instrumented sock for ease of application and safe, unobtrusive use [more details in Shah et al. (36)].

Immediately after testing in the clinic, subjects were asked to participate in daily life data collection and instructed on how to wear the instrumented socks and one Opal sensor over the lower lumbar area with an elastic belt. They were instructed to wear the sensors for at least 8 h/day for a week during daily life. Subjects removed the socks and the belt at night to recharge the batteries, and the data were stored in the Opal’s internal memory (8GB). After 7–14 days of data collection, the instrumented socks were returned by mail or in person, and the data were uploaded to a secure cloud-based database, then downloaded to a local computer for further processing using the same gait algorithms (after identification of appropriate length gait bouts) for both in-clinic and daily life gait assessments. Participants completed a usability questionnaire upon return of the devices.

### Measures of gait

The algorithms for extracting spatial and temporal measures of gait and turning are consistent across both laboratory and daily life settings, as described in prior studies (37, 38). For daily life gait analysis, the algorithm detects walking bouts using inertial sensor data from the feet and identifies turns based on pelvic yaw rotation (19, 37). Steps are grouped into walking bouts if the interval between them is no more than 2.5 s, and those with at least three steps lasting three seconds are processed using Mobility Lab’s commercial algorithms (APDM Wearable Technologies, Portland, Oregon) (39–41). The analysis algorithm employs the Unscented Kalman Filter to integrate accelerometer, gyroscope, and magnetometer data, precisely estimating the foot’s orientation and trajectory (42, 43).

To compare between in-clinic and daily life gait measures, we only assessed 17 metrics that were common between the in-clinic 2MWT and the at-home passive monitoring. For the home data, only participants who totaled at least 20 h of recorded data, spanning over at least 4 days, and encompassing at least 20 gait bouts were included. In the clinic setting, only participants who had at least 15 strides during the 2MWT were included. For daily life, 3 out of 12 enrolled subjects were excluded due to more severe disease, that precluded them from walking much at-home or resulted in them requiring assistive devices. Thus, 9 FRDA participants were analyzed for the comparison between the two settings. To restrict our HC population to be age-and sex-matched a propensity scoring algorithm was used to create a 1:1 comparative sample.

### Statistical analysis

Mann–Whitney U tests were used to compare between-group differences in subject characteristics, total weekly activity levels, and all gait measures. In addition, we computed Receiving Operating Characteristics (ROC) and calculated the Area Under Curve (AUC) (44) to discriminate FRDA patients and HC using gait metrics. Paired Wilcoxon tests were used to compare the laboratory and daily life gait measures within the FRDA and HC groups. All statistical analysis was performed using R Version 4.2.0 software. The statistical significance was set to *p* < 0.05.

## Results

### Group characteristics and activity levels

Eighteen people, 9 FRDA and 9 age-and sex-matched HC, were included in this analysis (we lost 3 out of 12 FRDA subjects due to wheelchair use at home which resulted in very few walking bouts). Table 1 shows the demographics and activity characteristics of subjects who participated in this analysis. For daily life analysis, a total of 99,216 strides across 1,008 h of recording were included. The FRDA group exhibited much lower levels of daily activity (including bouts/h, strides/h, turns/h, and median strides in a bout) compared to the HC group. For example, the median number of strides in a gait bout (representing bout length) was shorter in the FRDA group (13 [10, 14]) compared to the control groups (16 [15, 19]). The total number of strides averaged for gait characteristics over 7–10 days of daily life was 3,634 in the FRDA group and 12,870 in the control group. In contrast to daily life, assessment of gait in the clinic included, on average, 53 [52, 59] strides in the FRDA group and 57 [56, 62] strides in the control group.

**Table 1:** Demographics and weekly activity of each group.

	FRDA (*N* = 9)		HC (*N* = 9)		*p*
	Median	Q1, Q3	Median	Q1, Q3	
Age (years)	20	12, 48	18	13, 22	1
Sex (M, F)	5, 4	NA	4, 5	NA	1
GAA1	738	333, 758	NA	NA	1
GAA2	942	883, 1,008	NA	NA	1
Age at onset of gait unsteadiness	15	10, 36	NA	NA	1
Disease duration (years)	10	5, 12	NA	NA	1
Modified Friedreich’s Ataxia Rating Scale (mFARS) – Upright Stability Score	18	16, 19	NA	NA	1
mFARS total	30	28, 34	NA	NA	1
Total duration (hours)	85.82	76.19, 109.08	68.71	59.47, 69.75	0.0047
No. of days	10	9, 11	7	7, 8	0.0032
Bouts/h	1.6	1.24, 4.19	5.18	4.51, 8.73	0.0171
Strides/h	27.63	14.96, 58.03	169.7	107.87, 215.5	0.0006
Turns/h	4.71	1.99, 7	15.54	11.38, 20.68	0.0006
Strides in a bout	13	10, 14	16	15, 19	0.0018

Daily life activity measures compared with Wilcoxon Rank Sum Test (Mann–Whitney U). Gender compared using Fisher’s Exact Test.

### Clinic versus daily life gait measures discriminating gait between FRDA and HC

**Figure 2:**
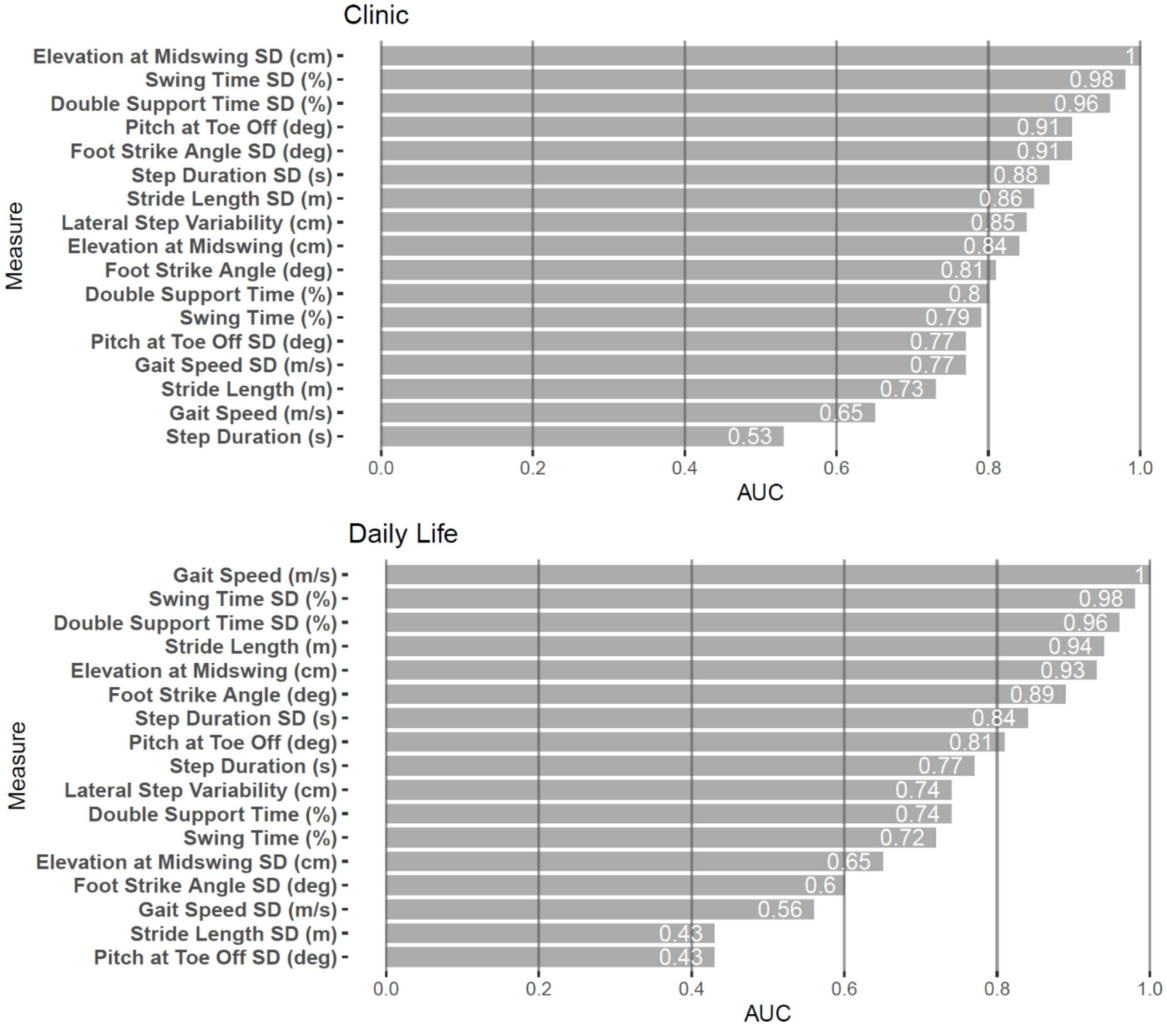
AUC plots (with 95% CI) for each gait measure in the clinic and daily life.

Different measures effectively discriminated FRDA from HC in the clinic versus daily life environments, but two measures performed strongly in both environments (see Table 2; Figure 2). The highest AUCs across both the clinic and daily life environments Swing Time SD (0.98 and 0.98) and Double Support Time (%) SD (0.96 and 0.96) were top performers. In the clinic, Elevation at Midswing SD also perfectly differentiated the groups with an AUC of 1.00 but it only performed with an AUC of 0.65 in daily life. Conversely in daily life, Gait Speed differentiated groups with an AUC of 1.00, but only performed with an AUC of 0.65 in the lab. Of the 17 measures tested, 11 gait measures had an AUC > 0.8 in the clinic whereas 8 gait measures had an AUC > 0.8 in daily life. Three timing variability measures (SD of swing time, double support time, and step duration) and 3-foot orientation measures (elevation, foot strike angle, and toe-off angle) were sensitive and specific with AUC > 0.8 in both environments.

**Table 2:** Median and IQR, AUC, and Wilcoxon *p*-values for each gait measure in the clinic and during daily life.

	Clinic				Daily Life			
Gait measures	FRDA (*N* = 9) Median [Q1, Q3]	HC (*N* = 9) Median [Q1, Q3]	AUC	Wilcox *p-*value	FRDA (*N* = 9) Median [Q1, Q3]	HC (*N* = 9) Median [Q1, Q3]	AUC	Wilcox *p-*value
Swing time SD (%)	**1.91 [1.72, 3.04]**	**0.81 [0.62, 0.97]**	**0.98**	**0.0002**	**4.26 [3.5, 4.47]**	**2.32 [1.98, 2.58]**	**0.98**	**0.0002**
Double support time SD (%)	**3.02 [2.24, 4.18]**	**1.43 [0.97, 1.82]**	**0.96**	**0.0003**	**6 [5.37, 6.46]**	**3.82 [2.85, 4.15]**	**0.96**	**0.0003**
Elevation at midswing (cm)	**1.87 [1.43, 3.37]**	**1.09 [0.89, 1.16]**	**0.84**	**0.0142**	**4.75 [3.92, 5.98]**	**3.36 [2.91, 3.87]**	**0.93**	**0.0012**
Pitch at toe off (deg)	**30.23 [27.07, 31.94]**	**40.7 [36.26, 42.2]**	**0.91**	**0.0019**	**23.54 [22.37, 27.6]**	**29.46 [28.27, 30.34]**	**0.81**	**0.0244**
Step duration SD (s)	**0.04 [0.03, 0.06]**	**0.01 [0.01, 0.02]**	**0.88**	**0.0075**	**0.09 [0.07, 0.12]**	**0.07 [0.05, 0.07]**	**0.84**	**0.0142**
Foot strike angle (deg)	**10.51 [6.54, 20.81]**	**21.34 [20.37, 21.59]**	**0.81**	**0.0304**	**14.95 [13.67, 19.24]**	**23.42 [21.52, 25.62]**	**0.89**	**0.0040**
Stride length (m)	1.04 [0.76, 1.15]	1.23 [1.16, 1.28]	0.73	0.1118	**1 [0.93, 1.14]**	**1.4 [1.31, 1.44]**	**0.94**	**0.0008**
Elevation at midswing SD (cm)	**0.92 [0.76, 1.35]**	**0.47 [0.4, 0.51]**	**1**	**0.0004**	2.29 [1.85, 2.53]	2.1 [1.63, 2.41]	0.65	0.2973
Gait speed (m/s)	1.08 [0.59, 1.18]	1.16 [1.05, 1.21]	0.65	0.2973	**0.93 [0.77, 1.06]**	**1.29 [1.24, 1.32]**	**1**	**<0.0001**
Lateral step variability (cm)	**6.04 [4.57, 6.72]**	**2.99 [2.69, 3.7]**	**0.85**	**0.0106**	8.13 [7.59, 8.91]	7.35 [6.83, 7.66]	0.74	0.0939
Double support time (%)	**22.82 [19.52, 34.26]**	**18.1 [17.28, 18.61]**	**0.8**	**0.0315**	23.25 [20.25, 24.87]	19.43 [19.01, 20.38]	0.74	0.0939
Foot strike angle SD (deg)	**2.91 [2.16, 3.75]**	**1.6 [1.52, 2.06]**	**0.91**	**0.0019**	6.85 [5.33, 8.76]	7.84 [7.17, 8.6]	0.6	0.4894
Swing time (%)	**38.56 [32.9, 40.28]**	**40.87 [40.63, 41.3]**	**0.79**	**0.0400**	38.33 [37.55, 39.94]	40.35 [39.83, 40.55]	0.72	0.1359
Gait speed SD (m/s)	0.08 [0.06, 0.12]	0.05 [0.03, 0.08]	0.77	0.0631	0.21 [0.2, 0.31]	0.26 [0.23, 0.27]	0.56	0.7304
Step duration (s)	0.55 [0.48, 0.58]	0.54 [0.51, 0.55]	0.53	0.8595	0.59 [0.56, 0.67]	0.56 [0.53, 0.58]	0.77	0.0625
Stride length SD (m)	**0.06 [0.06, 0.07]**	**0.04 [0.03, 0.06]**	**0.86**	**0.0114**	0.21 [0.18, 0.25]	0.22 [0.16, 0.24]	0.43	0.6665
Pitch at toe off SD (deg)	2.4 [2.01, 2.66]	1.91 [1.29, 2.08]	0.77	0.0625	5.14 [4.39, 5.4]	4.89 [4.67, 6.3]	0.43	0.6665

Bold indicates *p* < 0.05.

### Clinic versus daily life gait measures for each group

**Figure 3:**
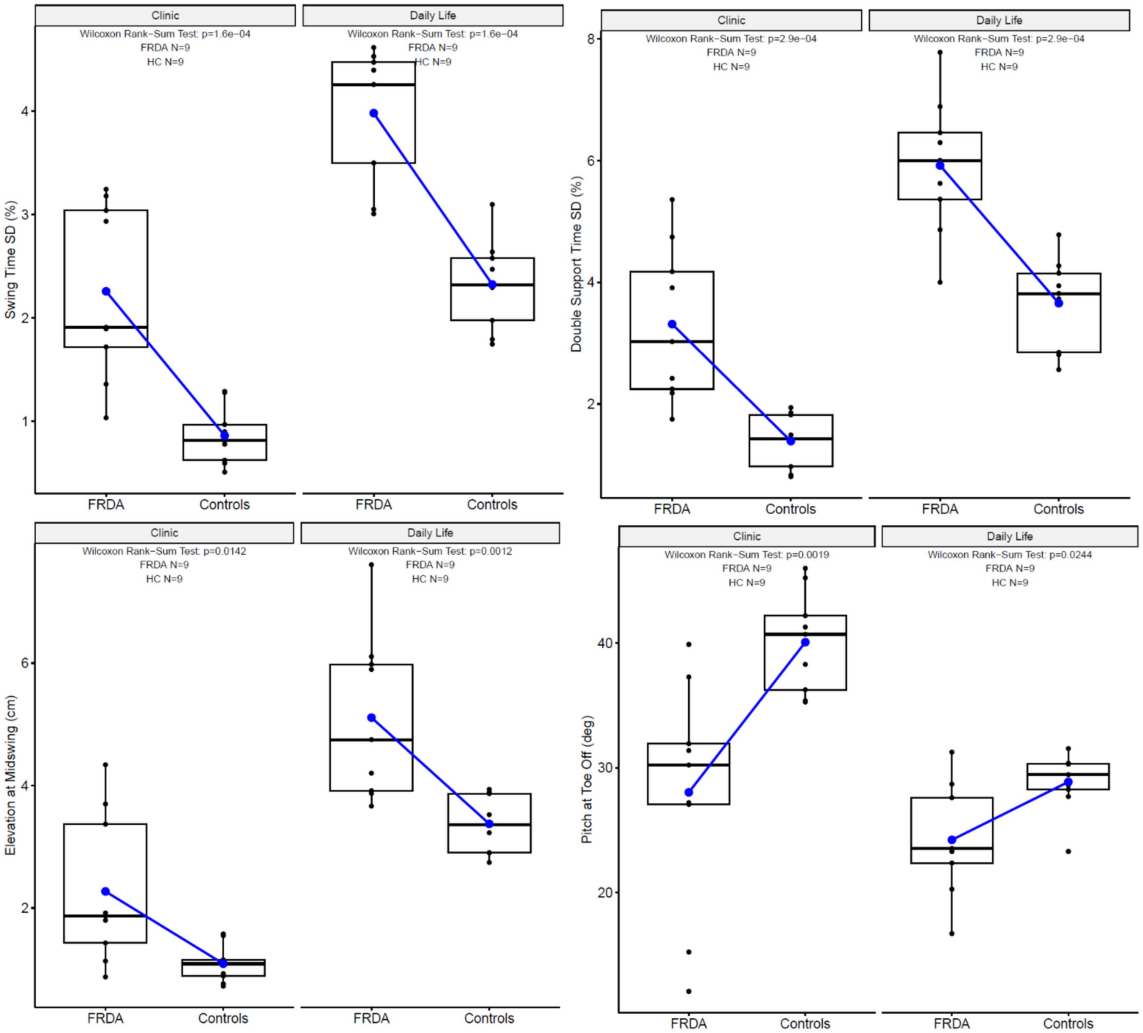
Box plots comparing the sensitivity of specific measures in-clinic versus daily life gait characteristics.

**Figure 4:**
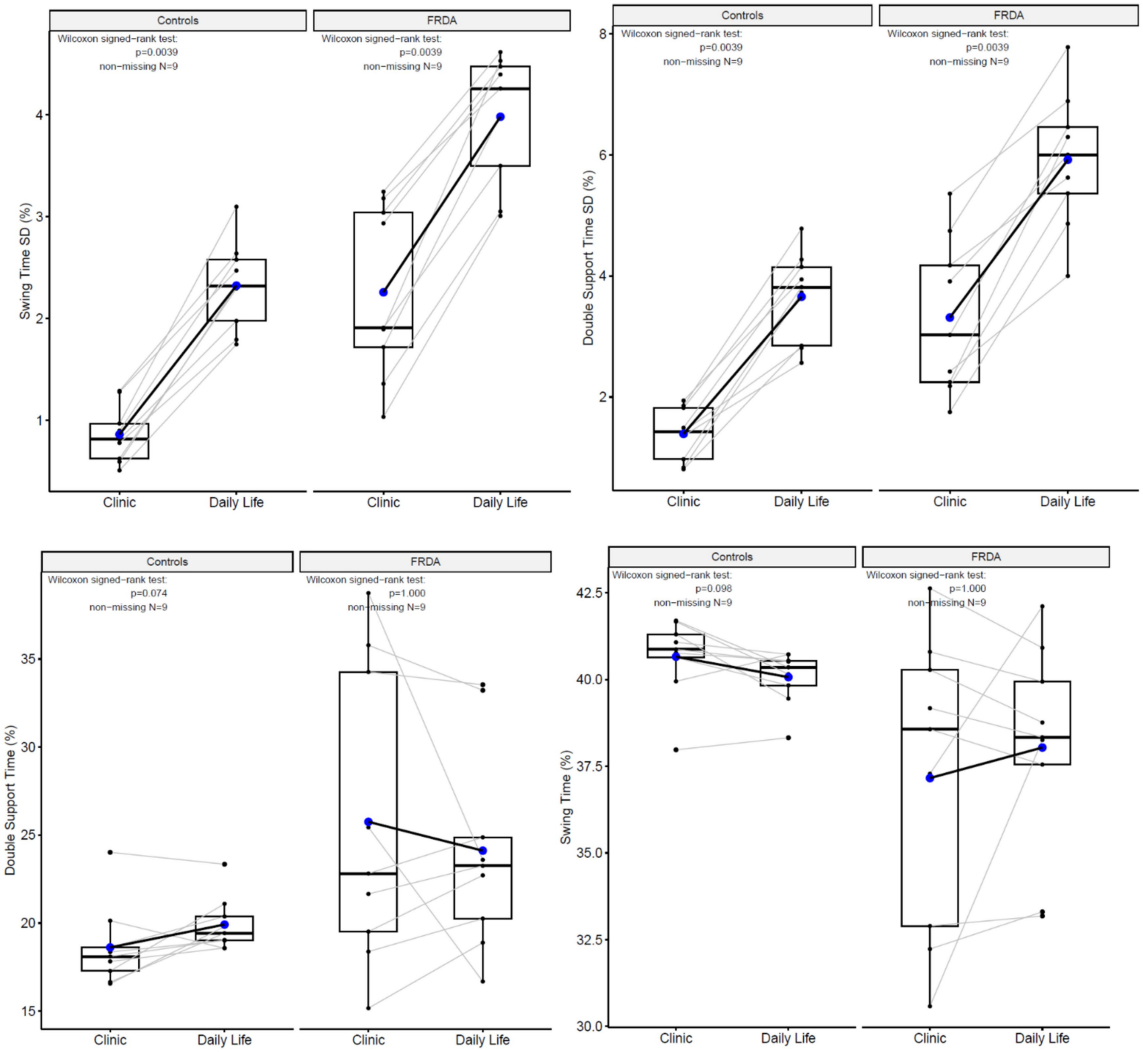
Paired box plots illustrating the most discriminating four measures differ in their values across environments.

Most of the gait characteristics differed across the two environments (Figure 3). Figure 4 illustrates the paired results of the top 2 discriminating measures in both environments, Double Support Time SD and Swing Time SD. Higher levels of variability are found for both cohorts in daily life compared to the prescribed gait in the clinic.

### Gait measures were significantly correlated with clinical measures

The top discriminative gait variability measures (Double Support Time STD and Swing Time STD) were also significantly correlated with mFARS US (*r* = 0.74 and *r* = 0.69) in clinic. in the FA population. These digital measures generally demonstrated stronger associations with clinical measures in the clinic compared to daily life (i.e., *r* = 0.74 and 0.69 in clinic vs. *r* = 0.68 and *r* = 0.45 in daily life with mFARS US). Additionally, these digital measures showed strong correlations with FARS ADL in the clinic (*r* = 0.50 and *r* = 0.53). Further, Elevation at Midswing in clinic was significantly correlated SARA total (*r* = 0.92), and strongly correlated with mFARS NE (*r* = 0.53), while Elevation at Midswing during daily life was strongly correlated with SARA total (*r* = 0.61) and mFARS NE (*r* = 0.58). In the SmartSox usability questionnaire, all participants indicated “agree” or “strongly agree” to statements indicating *I found it easy to learn how to use the SmartSox* and *I found the SmartSox system easy to use*.

## Discussion

This study aimed to identify the most discriminatory gait measures for use in clinical trials for FRDA from body-worn, inertial sensors during a 2-min, in-clinic walking at natural pace assessment and during a week of walking during daily life. In this study, the FRDA cohort exhibited significantly lower levels of daily walking and turning activity. We found that the most discriminative gait measures between participants with FRDA from HC vary between in the clinic and in daily life settings. Yet, two gait timing variability measures were sensitive and specific for FRDA in both settings. Except for these two gait variability measures, findings support our hypothesis that distinct gait measures best discriminate FRDA from HC in different settings.

Compared to the HC group, the FRDA group showed significantly reduced levels of daily activity. This included the number of walking bouts per hour, number of strides per hour, number of turns per hour, and number of strides in an average bout (Table 1). To qualify for enrollment in the larger study, participants must have been able to walk 10 meters, independently for up to 2-min for the prescribed, in-clinic assessment. As the majority of our FRDA cohort were early-and typical-onset patients with varying disease durations and levels of gait impairment, reduced daily activity compared to the HC group is expected (45, 46). This pattern of reduced ambulatory activity in daily life in young people with FRDA has been described previously (27, 29).

In this cohort, measures of gait that were most discriminative in participants with FRDA from the HC group usually, with 2 exceptions, differed between in-clinic and daily life environments. We found that 11 of the 17 measures tested in the clinic and 8 of the 17 measures tested in daily life had an AUC ≥ 0.8 (Table 2). The elevation of the feet at midswing standard deviation best discriminated participants with FRDA from HC in the clinic (Table 2; Figure 3). In contrast, gait speed best discriminated participants with FRDA from HC in daily life (Table 2; Figure 3). The consistency of the hard, level, clutter-free surface in the clinic, but not at home, may have allowed the increased variability of elevation of the feet (high stepping) to distinguish FRDA from HC. The likelihood for dual-tasking, distractions, and short gait bouts in daily life may have separated gait performance in FRDA from HC since FRDA is likely associated with shorter gait bouts, known to be related to slower gait, as well as less automatic gait, which depends upon higher levels of attention (21, 47). Alternative or additional factors maybe distinct motivational components, but in particular also the role of fatigue, one of the hallmark symptoms of FRDA (48). In clinical settings, the impact of fatigue on short ambulatory tasks (e.g., 25 feet or 10 meters) remains minimal, while extended ambulatory tasks (such as a 1-min walk), have a pronounced fatigue component. This suggests that daily living measures would have a significantly higher fatigue component than short in-clinic assessments (that do not force a longer walking task). It also is in-line with the differential discriminative performance of walking speed. Similar measures of in-clinic gait variability (toe-out angle, double support time, elevation of feet at mid-swing) have also been found to be sensitive to prodromal spinocerebellar ataxia, a related form of degenerative cerebellar ataxia (49).

Other studies of free-living, daily life measures of gait in individuals with FRDA have not drawn comparisons between body-worn, inertial sensors in daily life and body-worn, inertial sensors in-clinic (27, 29). However, one study compared daily life measures to gait metrics derived from a 7-meter GAITRite^®^ Walkway System in laboratory environments (28). This study found the preferred speed base of support, preferred speed base of support variability, and fast speed base of support variability to be normally distributed (28). Yet, the researchers argued that daily life gait measures alone are limited by large variability as determined by distance walked, step count, and duration of activity (28). Research in other degenerative cerebellar ataxias also utilizing APDM Wearable Technologies agree that assessments during daily life walking conditions yields much greater variability compared to lab-derived gait metrics (26). Daily life monitoring may capture hallmark features of cerebellar disease like turning movements or other gait characteristics that otherwise would not be fully captured in a controlled, clinical setting (26). Daily life monitoring studies on gait impairment in Parkinson’s disease and Multiple Sclerosis also demonstrate an increase in gait variability when recorded during free walking in daily life compared to a prescribed walking task in the clinic (6, 9). However, comparisons of gait metrics collected at home versus in the clinic are complicated because gait bouts in daily life tend to be much shorter than in clinical tests and gait bout lengths influence gait metrics (6). Given these findings, we suggest that gait measures collected in the home environment can provide valuable and complementary information to the prescribed walking tasks collected in the clinic.

We found two parameters that performed best in both cohorts in the different environments: the variability of swing time and the variability of double support time. In contrast, one study found that of 15 gait parameters measured in daily life over the span of 6 days, swing and stance durations were most discriminative between early-onset FRDA and control groups (27). However, this study found relatively low intraclass correlation coefficient due to the nature of collecting continuous data in daily life and limited walking of the FRDA participants (27). Yet, these authors provide a compelling argument that digital measures of gait provide the opportunity to assess specific gait characteristics and may offer greater sensitivity to evaluate motor function (27).

The recent FDA approval of omaveloxone for FRDA patients (50) has heightened the desire for quantitative, clinical performance outcomes for measuring the effectiveness of interventions over time while reducing the size of clinical trial cohorts. To improve the success of these trials, it is crucial to develop biomarkers and clinical outcome measures that continuously assess disease severity, disease progression over time, and treatment effectiveness. Wearable inertial sensors in-clinic and in daily life can provide sensitive and complementary assessments of ataxia and in turn, improve upon the current standard of natural history studies and interventional trials.

The current study is limited by size and duration. This study included only 9 ambulatory, early-stage participants diagnosed with FRDA and 9 matched healthy controls tested in the clinic, followed by free-living in daily life. FRDA enrollment in the study was limited by a variety of factors, including school and recreational schedules of younger participants, the COVID-19 pandemic, and enrollment in drug trials. No direct reports were collected from parents or caregivers in this study, which may be helpful in future studies to capture additional context that would not otherwise be captured in a clinical setting. This study did not address the ability of gait measures to monitor disease progression over time. Future studies should add longitudinal testing for future trials aimed at slowing the progression of FRDA. To confirm the validity of the results, future studies are needed with larger groups for both early-onset and late-onset FRDA to ensure that the findings can be applied to the broader population and determine potential discriminative ability between the two groups.

This study has identified a set of objective and discriminative gait measures from body worn inertial sensors collected during free living in daily life and during a prescribed 2-min walk in the clinic. The variability of gait timing measures was sensitive and specific for FRDA in both daily life and the clinic, but distinct digital gait characteristics were found to be best for each of the two environments (gait speed and variability of foot elevation, respectively). Future research involving tracking disease progression, validity, and reliability of a larger cohort of people with FRDA is needed to identify the most useful digital gait biomarkers for clinical trials.

## Data Availability

The datasets presented in this article are not readily available because de-identified gait and clinical data will be made available with the sponsor and PIs’ consent. Requests to access the datasets should be directed to Vrutang Shah, shahvr@ohsu.edu.
